# Non-Conventional Deformations: Materials and Actuation

**DOI:** 10.3390/ma13061383

**Published:** 2020-03-18

**Authors:** Bruno Vermes, Tibor Czigany

**Affiliations:** 1 Department of Polymer Engineering, Faculty of Mechanical Engineering, Budapest University of Technology and Economics, Műegyetem rkp. 3., H-1111 Budapest, Hungary; vermesb@pt.bme.hu; 2 MTA-BME Research Group for Composite Science and Technology, Műegyetem rkp. 3., H-1111 Budapest, Hungary

**Keywords:** morphing, shape adaptation, coupling, composites

## Abstract

This paper reviews materials and structures displaying non-conventional deformations as a response to different actuations (e.g., electricity, heat and mechanical loading). Due to the various kinds of actuation and targeted irregular deformation modes, the approaches in the literature show great diversity. Methods are systematized and tabulated based on the nature of actuation. Electrically and mechanically actuated shape changing concepts are discussed individually for their significance, while systems actuated by heat, pressure, light and chemicals are condensed in a shared section presenting examples and main research trends. Besides scientific research results, this paper features examples of real-world applicability of shape changing materials, highlighting their industrial value.

## 1. Introduction

Making materials multifunctional by endowing them with additional features, such as self-healing [1,2] or integrated health monitoring [3,4] is one of the greatest endeavors of today’s material science. The additional feature can also be a special mechanical behavior: morphing. Materials that change shape non-conventionally (morphing materials) allow us to design and manufacture structures that work more efficiently than their conventional counterparts, therefore their research and development are of primary importance for not only academia but industry as well (e.g., the energy and transportation industries). By definition, in this paper, conventional deformations are induced by loads of the same kind as the deformation itself (e.g., extension in response to tensile loading or bending in response to bending moments). Hence, non-conventional deformations are induced by dissimilar loads (e.g., bending in response to tensile loading or twisting in response to bending moment) or actuated by means other than mechanical loading (e.g., deformation to electricity, heat, pressure, etc.)

There is a wide variety of morphing concepts in the literature, and they can be categorized by a number of principles. The main sorting principle of this paper is the type of actuation that is needed for the shape change to take place. The most common means of actuating shape changes are electricity, heat, pressure, light, chemicals and mechanical loads. These concepts can be further categorized as active, semi-active and passive, depending on the extent of human (active) and environmental (passive) influence. There are no clear boundaries between these categories, although electricity is more a kind of active actuation as it can be controlled almost independently of operating conditions, while mechanical loading is set by the operating conditions in the case of a structural part. Heat is a semi-active actuation, being greatly dependent on the environment but very artificially controllable at the same time. 

The optimal shape-changing concept always depends on the application. In some cases, we do not want the material to adapt to its environment; instead, we wish to control its deformation actively. In these cases, systems actuated by electricity [5], heat [6] or pressure [7] may be considered. On the other hand, there are situations when environmental adaptation is what we are after to achieve maximum working efficiency of a structural part. In the case of marine or wind turbine blades, for instance, their shape for optimal energy yield depends on the direction and speed of the flowing fluid, i.e., the mechanical loading the blades are subjected to [8]. A mechanically actuated shape-changing material could passively modulate its shape (e.g., twisting) in response to changing loads (e.g., bending moments), resulting in significant efficiency gains.

This review aims to introduce some selected shape-changing concepts from the literature categorized by the type of actuation giving an up-to-date overview of this field of research.

## 2. Electro-Actuated Shape Adaptation 

In this review, electro-actuated shape adaptation is defined as controlled deformation or resistance to deformation in response to applied electricity. Electricity is relatively easily controllable independently of environmental conditions and can be exploited to actuate non-conventional deformation in various ways. It can power electromotors [9,10,11,12,13] as well as piezo- [14,15,16,17,18,19] and other electrosensitive materials. Due to this diversity, there is an extensive literature on electro-actuated systems. This section discusses some selected concepts.

Electric motors are widely used in motorsport, and industries like the transportation or the aerospace industry, although seldom for morphing applications. The aerospace industry shows the greatest interest in motor-actuated morphing concepts. In a broad sense, conventional wing flaps are morphing structures themselves; however, there are concepts where the triggered deformation is less evident, therefore they are more easily categorized as morphing structures. Garcia et al. [9] designed a micro air vehicle in which torque rods placed along the flexible membrane wings and connected to a servo motor were responsible for control authority by operating roll maneuvers. Stanford et al. [10] investigated a similar structure with asymmetric twisting of the wings with the addition of numerical (static structural and aerodynamic) modelling and genetic algorithm-based optimization. With these tools, they developed a design that showed significantly improved roll-rate and lift-to-drag ratio compared to their baseline design, highlighting the importance of computer-aided optimization, even in the case of simple mechanism actuated systems. Ahmed et al. [20] introduced an aerodynamic optimization process to find the optimal anti-symmetric wing twist distribution of a micro air vehicle (MAV) to achieve improved roll performance together with a low level of produced drag. Their optimization tool showed a rapid convergence to the optimal solution for wing twist design. Motors can be used to achieve deformations other than twisting, too. Boria et al. [11] used genetic algorithm-based hardware-in-the-loop optimization for camber morphing of a composite wing skin actuated by a single servo motor. A system like this can significantly modify the camber and therefore the aerodynamic characteristics and efficiency of the wing with relatively little effort. The effort needed for shape adaptation can also be decreased by altering the mechanical properties of the material for the duration of the deformation. Hamilton et al. [12] reduced the stiffness of the matrix material with a temperature controller by heating it up for the morphing phase and then increased its stiffness by cooling the material back to preserve the new shape. Hybrid approaches like this may require multiple actuation systems, but their energy efficiency or the achievable greater deformations can justify their use in many cases. Another way to increase efficiency is to decrease the number of actuators (motors) needed for complex morphing. Winstone et al. [13] proposed a design of a single-motor-driven worm robot that was capable of peristaltic locomotion thanks to the design of its segments enabling complex motions to simple impulsions.

Besides electromotors, electricity can be exploited for morphing applications with materials that are electrically responsive themselves. Piezoelectric materials are one of the most researched electroresponsive materials as they reliably convert electrical energy into mechanical energy and vice versa [14]. Although they generally exhibit relatively low actuation strains, their high force output, even at high frequencies, makes them good candidates for not only vibration dampening but also as actuators to alter the shape of attached materials. Furthermore, Fichera et al. [15] showed that with proper design, deflections can be significant, too, without sacrificing the frequency of response. Yoon et al. [16] developed a camber morphing control fin with integrated piezo-composite actuators in its skin. These structures are generally actuated by a servo motor, but piezo-actuation can simplify the structure; there is no need to convert rotational motion to linear actuation. However, piezo-actuators increase the weight of the morphing structure. For the best weight-to-performance ratio, the optimal number and size of actuators need to be used for a certain application. These can be found by numerical optimization [17]. Jodin et al. [18] investigated a hybrid system, where the camber of a wing was altered by shape memory alloys (SMAs), while trailing edge vibrations were controlled by piezo-actuators, providing some aerodynamic benefit over a static trailing edge. In complex morphing structures, piezo-materials are often complemented by other means of actuation for multifunctionality. Bye et al. [19], for instance, designed a morphing airplane that can significantly change the shape of its wings to adapt to different flight scenarios (e.g., cruising or high-speed dash), for which they employed thermopolymers and shape memory polymers (SMPs) besides piezo-actuators. Nabawy et al. [21,22] developed a comprehensive analytical model to provide a mapping between force, displacement, charge and voltage for piezoelectric actuators. They also validated the model against experimental results. The significance of their work lies in making the performance of piezoelectric actuators predictable in dynamic operations and analyzing the dynamic electromechanical coupling factor, which defines the exchanged electrical and mechanical energy in a single piezoelectric flexure cycle. Based on these results, the applicability of piezoelectric actuators can be assessed for different use cases (e.g., morphing or flapping wings).

Electroactive polymers usually have the advantage of being flexible, lightweight and relatively easy and inexpensive to manufacture. Dielectric elastomer actuators (DEAs) work by the principle of having a flexible membrane between two electrodes that are squeezed together by the Coulomb force when placed under high voltage current. DEAs can demonstrate large strains of a few thousand percent [23], but they can be electromechanically unstable. Kornbluh et al. [24] achieved strains exceeding 200% with response speeds up to 2000 Hz with DEAs. Duduta et al. [25] proposed a DEA system with carbon nanotube electrodes to increase the peak energy density to similar levels as the energy density of human muscles, overcoming one of the main limitations of this approach. Ferroelectric polymers change their polar state in response to the applied electric field, and this causes strain, but their energy density is relatively low. Nevertheless, these polymers can be incorporated into nanogenerators for energy harvesting [26]. 

**Figure 1:**
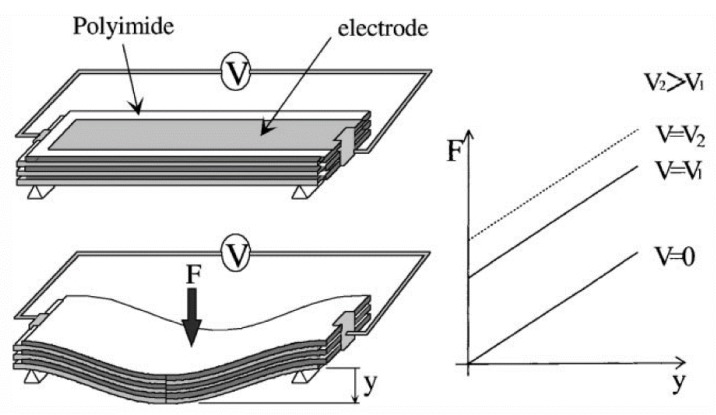
Electro-bonded laminate working principle [31].

The working principle of electro-bonded materials is that instead of a permanent, constant adhesion between the constitutive layers, the strength of the interlaminar connection is a function of the applied voltage (Figure 1). Instead of deforming the material, electricity makes the bending stiffness of the structure reversibly variable, therefore controlling its deformation when loaded. Heath et al. [27] more than doubled the bending stiffness of a sandwich structure by electro-bonding the two halves of the core material with 4 kV. Bergamini et al. [28] achieved an 18-fold increase in bending stiffness with a similar approach. Heath et al. [29] also investigated interlocking electro-bonded layers where the interfaces were not plain but followed a cosine wave form. This way they achieved direction-dependent variable stiffness. Testoni et al. [30] proposed a concept similar to the previously mentioned electro-bonded approaches but with a twist. They placed truss-like mechanical switches between composite skins as the core material that had a threshold in their actuation power (for axial sliding) when electricity was applied thereby modifying the mechanical behavior of the loaded sandwich structure.

**Figure 2:**
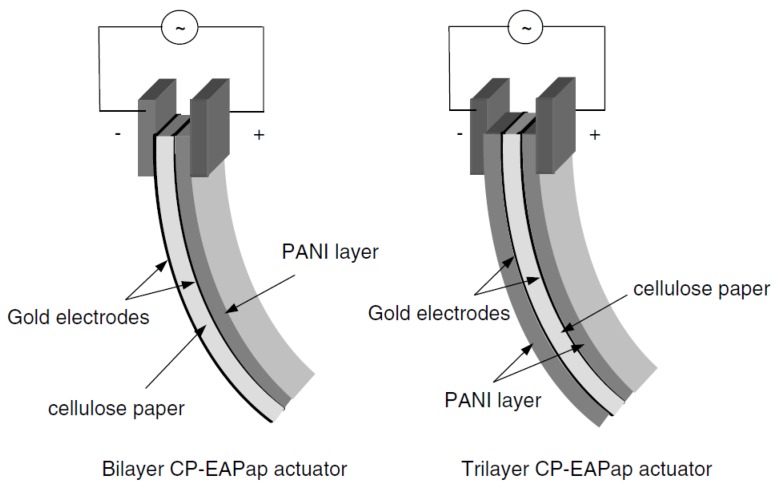
Bi- and trilayer polyaniline coated electroactive paper actuators [32].

Conjugated polymers can be made electrical conductors by a process called “doping” when the molecule is oxidized or reduced. Voltage can change their oxidation state leading to dimensional changes. Advantages include light weight, low actuation energy and relatively high energy density. Polyaniline [32] and polypyrrole [33] are the most researched conjugated polymers for morphing applications, primarily due to their significant strains to an applied voltage. Figure 2 illustrates the schematic of a bi- and a trilayer polyaniline coated electroactive paper actuator [32].

Not only solid-state materials can demonstrate deformation to electricity, but gels and fluids, too. Ionic gels can usually deform (bend) significantly in response to low actuation voltages due to counter-ion osmotic pressure. Various types of ionic gels exist, but Bucky-gels are among the most advanced ones as they can operate without an external ionic solution. In response to voltage, anions and cations from the internal polymeric electrolyte film separate and move towards the opposite electrodes. As the two different ions differ in size too, they occupy unequal space near the outer layers leading to an internal pressure difference and therefore the bending of the material [34]. A limitation of the concept is the back-relaxation even when voltage is maintained [35]. Non-ionic gels exist too and can behave similarly to how ionic gels do (e.g., quick, large deformations), but they require much higher actuation voltages [36]. Some hydrogels can even demonstrate bidirectional bending behavior [37]. Electrorheological fluids can change their viscosity in response to applied electricity by forming oriented chains of dielectric particles in an insulating fluid. The application of this easily controllable system varies from stroke rehabilitation robots in medicine [38] to active suspension systems in transportation [39]. 

Other electro-actuated morphing concepts exist as well, such as electro-origami systems consisting of simple electrodes, an insulator and a liquid dielectric that are capable of muscle-like mechanical performance in response to applied electricity [40].

Table 1 contains some important data about selected electro-actuated morphing systems. Modelling and experimental results are compared in the table only where they significantly differed. This approach applies to each table in the paper.

## 3. Shape Adaptation Actuated by Heat, Light, Pressure and Chemicals

This section discusses shape-changing material concepts where actuation is realized either thermally, chemically or by applied light or pressure. 

The simplest thermally actuated morphing materials exploit thermal expansion. Bimetals, for instance, consist of two different metals attached together. As the coefficients of thermal expansion for the two metals differ, they elongate at a different rate in response to applied heat leading to a bending moment and thus deformation of the structure. Pre-buckled bimetals can even be used as heat engines due to their thermo-mechanical instability (snap-through energy release) [41,42]. 

Shape memory alloys (SMAs) deform as a result of solid phase transitions. When cooled down, the so-called twinned martensite phase of the material can be deformed by loads. When heated, the material enters a cubic, symmetric austenite phase, regaining its original shape [43]. Kim et al. [6] developed a nickel titanium (NiTi) alloy coil spring actuator that can be used as an artificial muscle due to its high energy density contraction in response to applied heat. Shaw et al. [44] even demonstrated that NiTi honeycomb structures exhibit not only significant shape memory behavior but a high level of superelasticity as well, when loaded in-plane. Although most SMA research focus on NiTi alloys, there are other materials showing similar behavior, too. Ogawa et al. [45], for instance, introduced a magnesium alloy that has similar properties to conventional NiTi SMAs but for only two-thirds the weight.

**Figure 3:**
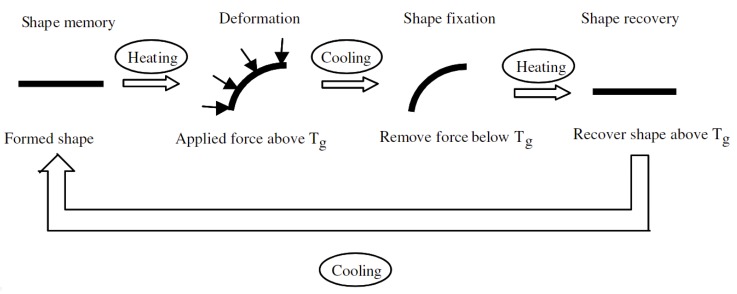
Schematic working principle of shape memory polymers [52].

Shape memory polymers (SMPs) are similar to SMAs in the sense that they are able to regain their original form in response to external stimuli (Figure 3). Although there are SMPs that are excited either electrically (Joule heating) [46] or by light [47], most of these polymers are actuated thermally. SMPs are cross-linked polymers with so-called transition segments between links. When heated above the transition temperature (either T_g_ or T_m_ depending on crystallinity), the transition segments display a drop in stiffness therefore the material becomes easily deformable, but the cross-linked structure hinders translations. Subsequent cooling fixes the deformed shape, and the original shape is regained through elastic recovery when the material is heated again. Some SMPs can elongate up to ten times their original lengths prior to failure [48], while others can have recoverable strains of 800% [49]. This makes these materials extremely useful where high deformations are needed (e.g., biomedicine), unlike SMAs, which have approximately 8% maximum strain [50]. The shape memory effect can also be exploited to produce artificial muscles with coiled geometries [51]. Despite the outstanding recoverable strain capacity, the applicability of SMPs is limited due to their low strength and recovery stress. To overcome this limitation, SMPs are often used as a matrix of composites. The most common reinforcements are fibers [52] and carbon nanotubes [53]. The latter is useful for Joule-heating, too.

Heat can trigger the deformation of so-called liquid crystal polymers, too. The deformation is a consequence of a crystalline to amorphous phase transition by the realignment of mesogens. During the initial programming of the material, mesogen alignment can be achieved in various ways (e.g., electric fields or mechanical loading), after which crosslinking the polymer finishes the process. Heat causes the mesogens to get disoriented, leading to often large deformations [54,55]. Yang et al. [56], for instance, demonstrated 300% to 400% reversible contractions of micron-sized liquid crystal elastomers.

**Figure 4:**
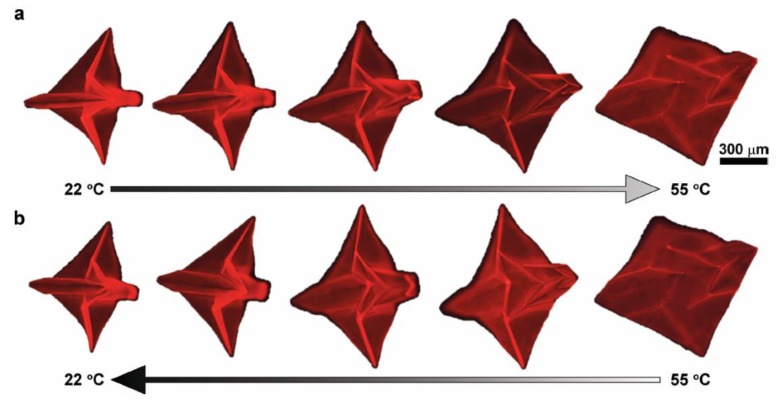
Thermally actuated origami folding: (**a**) cooling, deswelling and flattening, (**b**) heating, swelling and folding [58].

Hygromorphic materials work by the principle of controlled water swelling, but the swelling of some of these systems greatly depends on the temperature. At a certain temperature, some hygromorphic materials change from a swollen hydrophilic phase to a shrunk hydrophobic one [57]. With a tri-layer structure, Na et al. [58] demonstrated large deformations of a flat sheet to applied heat. The deforming structure could reversibly form an origami bird (Figure 4), the response time being the limitation of the approach with a few minutes required for total folding.

Although light-actuated shape changing materials might not be as extensively researched as electrically or thermally actuated systems, several different photoresponsive concepts exist due to their advantages, such as the good controllability and focusability of the actuating light. The precise, local stimuli can even allow for nanoscale shape adaptation responses. Irradiation usually leads to a reversible change in at least one property, such as the shape of the material [59]. Besides or instead of actuation by heat, some SMPs and liquid crystal polymers can be actuated by light, too. In most cases, photoactive SMPs contain photoreactive molecules to ensure photoresponse [47,60], but a nanocoat layer on the surface of a simple thermally active SMP can also imbue it with photoactivity [61].

Photoresponsive liquid crystal polymers usually contain azobenzene fillers to operate. Most of these actuators are thin films showing bending deformations. This is due to the primarily superficial effect of the light that contracts the outer layer causing the film to bend. These actuators are relatively easy and inexpensive to manufacture, but their thermal instability and often long response times limited their applicability for a long time [62]. Recently, however, Zeng et al. [63] demonstrated a photoactive liquid crystal polymer with a response frequency of almost 2000 Hz, overcoming one of the main disadvantages. Photoresponsive gels exist too, but they are often actuated through photothermal effects rather than purely by light. Wei and Yu [64] achieved photo-actuated contraction of 70% of a thermally sensitive hydrogel with azobenzene. However, that change in volume took approximately 60 minutes, showing one of the main disadvantages of these materials once again.

Pressure is most commonly used for actuation in the conventional piston-cylinder setup. Depending on the pressurized medium, pressure actuated systems are usually categorized into two groups: pneumatic (compressible fluid) and hydraulic (practically uncompressible fluid). Although the microscaling of these systems is a challenge due to the required low-friction microseals, piston-cylinder microactuators do exist [65]. 

Numerous other pressure-actuated concepts have been proposed in the past decades. Most of them can be categorized into a group called elastic fluidic actuators (EFA). Besides EFAs, drag-based actuators also exist. These use flowing fluids and exploit their drag force. Due to their working mechanism, these actuators usually operate smoothly but produce only moderate actuation forces [66].

EFAs can be further divided into sub-categories such as membrane [67], balloon [68] and bellow [69] types and artificial muscles [70], of which the first two types are the most common. Membrane actuators are usually elastic sheets, which can be expanded by pressure. Membranes with corrugated surfaces are often used to grab objects [71]. One of the main limitations of membranes is the limited deformation they are capable of. Balloon actuators, on the other hand, are much more versatile in terms of achievable deformation and can produce non-conventional deformations, too. 

**Figure 5:**
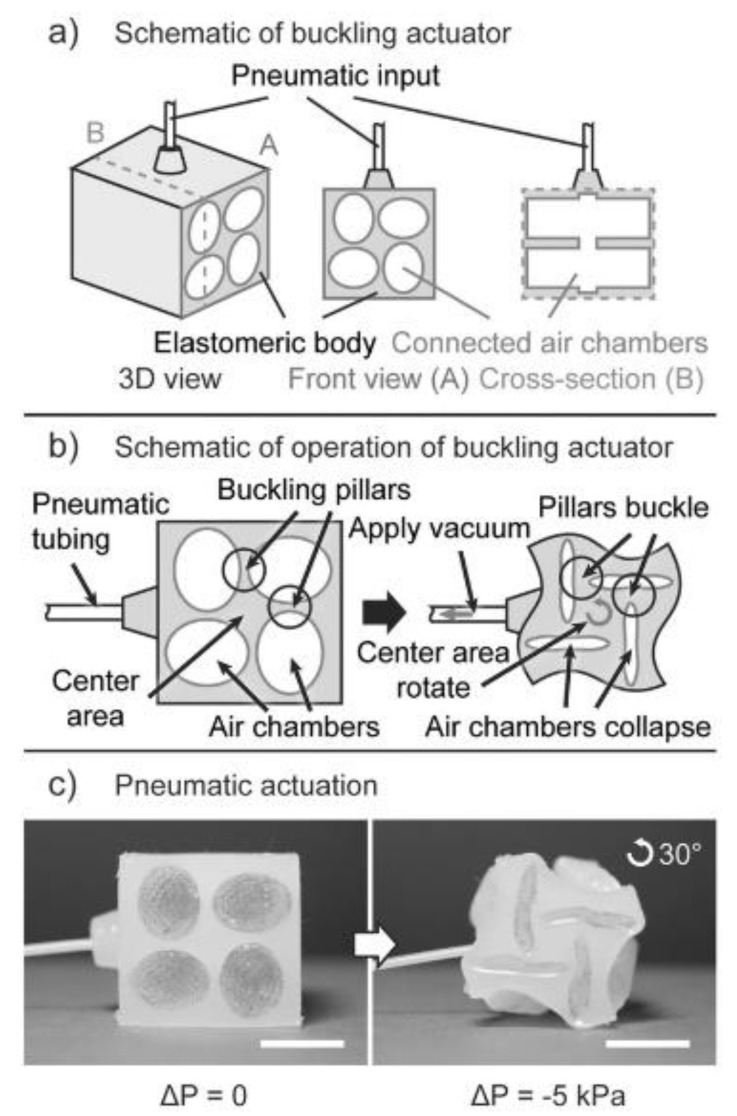
Schematics of a pneumatic buckling actuator. (**a**) actuator structure, (**b**) actuator operation, (**c**) pneumatic actuation [73].

Balloon-type actuators can bend, twist or extend/contract when pressurized, depending on the design of the actuator. Gorissen et al. [72] achieved large bending deflections of an elastic cylinder with a pressurizable eccentric inner void going along its length. The simple working principle is that the line of action of the applied force is offset from the neutral axis of the cylinder when the internal void is pressurized, leading to a bending moment and the bending of the whole structure. Ikeuchi et al. [68] achieved pressure-activated bending, too, but with a slightly different approach. They developed a micro-membrane with one flat side and the other side consisting of connected micro-chambers in length-section view. When internal hydraulic (water) pressure is increased, the chambers would expand leading to bending in the opposite direction. The concept was shown to be suitable for miniaturization, which is often a major concern in medical applications, for instance. It is also possible to achieve bending by connecting blocks with inflatable balloons. This way, segments can “bend” relative to each other when pressure is applied [7]. Moreover, based on very similar mechanisms to the ones just discussed, torsion [73,74] and extension or contraction [75] can be achieved, where the mode of deformation mainly depends on the design and position of the pressurizable chambers (Figure 5).

Shape changes can be actuated chemically, too. The simplest concepts build on controlled swelling and usually do not require any chemical reaction. These still count as chemical actuation, as the actuator is a chemical agent, usually water. As mentioned before, hygromorphic materials can change between a hydrophilic and a hydrophobic state with temperature, therefore, they are practically chemically actuated systems to some extent [57]. One of the most researched concepts builds on the biomimetics of the sea cucumber dermis. It is possible to manufacture a nanocomposite system that consists of percolating nanofibers with hydrogen bonds between them, making the structure stiff. However, when water is added, the structure loses the majority of its stiffness due to the competitive bonding effect where water–fiber bonds prove to be stronger than fiber–fiber bonds [76,77]. This way, water can significantly influence the stiffness and therefore the shape changing capability of the structure. The pH level of the surrounding medium can cause changes in dimension and shape, too, typically through the ionization of certain functional groups [78]. 

Table 2 lists some of the shape-changing concepts actuated by either heat, light, pressure or chemicals.

## 4. Mechanically actuated shape adaptation

Non-conventional shape changes can be actuated by mechanical loads, too. A typical limitation of these systems is that the deformations are usually difficult to control independently of the working conditions, especially in the case of structural parts that are inevitably under inconstant mechanical loads during operation. On the other hand, this “passive” actuation approach can be extremely advantageous when the operational mechanical loads are predictable and the optimal shapes to the various loading scenarios are known. For instance, the optimal angle of attack of turbine blades changes with wind speeds and a blade structure that would twist the right amount in response to increasing aerodynamic (bending) loads can significantly increase energy efficiency [81].

The mechanical behavior of structures greatly depends on their internal architecture. Mechanism-based shape-changing concepts often achieve controlled deformations by introducing changes to the internal architecture, leading to altered mechanical characteristics of the whole structure. Ajaj et al. [82,83] developed a wing-box capable of torsional stiffness adjustments with simple internal mechanisms. They tuned torsional stiffness by translating the span-wise spar webs closer to or further from the shear center of the cross-section of the wing-box. Greater distances from the shear center led to increased resistance to torsion, and the authors even identified optimal spar positions for three different flight scenarios by carrying out an optimization study. Runge et al. [84,85] proposed a different method to achieve controlled twisting by modifying the position of the shear center of an airfoil. They used clutch-like mechanisms to control the relative movement of internal discontinuous walls in a box section. When they released the clutch, the two halves of the discontinuous walls slid on each other, generating significantly less resistance to twisting than when the walls were fixed by the clutch. This way, they were able to control the twist angle of the wing section. This method, however, was still not absolutely “passive” as it needed external energy for actuation.

**Figure 6:**
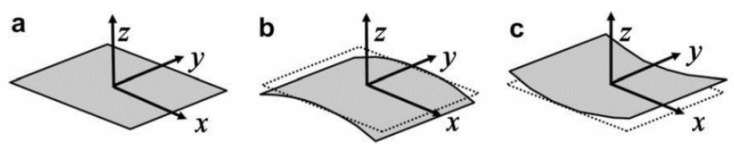
Bistable laminate (**a**) reference flat plate, (**b**) and (**c**) stable geometries [89].

Other mechanically actuated shape-changing concepts utilize the bi- or multistability of materials or structures. These systems have at least two equilibrium states (and shapes) (Figure 6). Often there are large displacements between the distinct equilibriums, and the actuation energy needed for the snap-through between these states is relatively low. A limitation of these systems is the stepwise deformation—a threshold load activates the “mechanical switch” and results in a big jump in deflection, practically instantly. Most often, bistability is achieved with composites that have asymmetric layups. Because of the asymmetry, the thermal stresses do not cancel each other out in the out-of-plane direction when cooled down from the manufacturing temperature. This leads to warping and bistable behavior in some cases. Another similar approach does not build on asymmetry but on prestressed laminae in a symmetric composite layup. Prestressing leads to stress fields similar to those in asymmetric composites. The main advantage of the prestressing approach is the higher achievable snap-through force [86,87,88]. 

Daynes et al. [90] developed an aeroelastic rotor blade with a bistable trailing edge. They used several internal laminates in the flap, where some laminae of each laminate were prestressed before and during the curing cycle. The mechanical behavior of the bistable flap was set by the properties and placement of the internal bistable laminates and a 10° snap-through deflection was achieved. The magnitude of the deflection and the snap-through load can be tuned by the layup, material properties, number and placement of the constituting laminates, as well as by the magnitude of the prestressing, making the aerodynamic characteristics of the bistable structure tunable. Furthermore, with the right geometrical design, it is possible to achieve tristability with only bistable laminates [91].

One of the most promising mechanically actuated shape-changing concepts builds on the mechanical coupling between loads and deformations of different modes (e.g., twisting in response to bending loads). Composites with special layups can demonstrate such mechanical coupling behavior. Historically, fiber-reinforced composites are mainly used for their outstanding specific mechanical properties, but mechanical coupling can give them the multifunctionality they need to further spread as advanced structural materials. 

In the case of composites, mechanical couplings are most often visualized as a set of coupling parameters in a 6x6 (so-called ABD) matrix, which establishes the relationship between six types of loads and six types of deformation (extension, transverse extension, in-plane shear, bending, transverse bending and twisting). This model is called the Classical Laminate Theory (CLT), and it is the simplest analytical model to characterize the mechanical behavior of composite laminates. The CLT builds on some assumptions (or simplifications), such as assuming plane stress state, linear elasticity and perfect interlaminar bonding, but these simplifications do not lead to major inaccuracies for thin laminates with small deformations. On the other hand, the low computational weight of the model and the fact that it quantifies each constituent of the coupling behavior with a single number for a given laminate allow for relatively simple design, evaluation and optimization. York [92] categorized and characterized composite laminates based on their coupling behavior. He found that the 3x3 extensional (A), bending (D) and extensional-bending (B) matrices can be populated (with non-zero terms) in only a few different ways, which depend on the layup of the composite. Moreover, any of the aforementioned six loads can lead to any of the six types of deformation. The only question is the magnitude of the coupling effect and how the layup needs to be designed to achieve the selected kind of coupling. York also published papers individually dedicated to extension-shear [93], extension-twist [94] and bend-twist [95] coupled composites with design recommendations for each case.

Extension-shear coupling is amongst the least researched coupling modes, although it has been demonstrated that it can produce significant and useful bend-twist coupling by strategically placing the laminates on a wing-box [93]. Li et al. [96] even demonstrated that hygro-thermal shearing distortion of extension-shear coupled laminates can be reduced, which might lead to an increased research interest in these laminates in the future.

Extension-twist coupling can be exploited in helicopter rotor blades, for instance. At higher rotational speeds, extensive loading becomes greater and leads to increasing twisting deformation of the blades. This way the blades can produce more lift, making the vehicle more efficient [94,97]. Similarly to extension-shear laminates, extension-twist laminates can be designed to be hygro-thermally stable, too [98].

**Figure 7:**
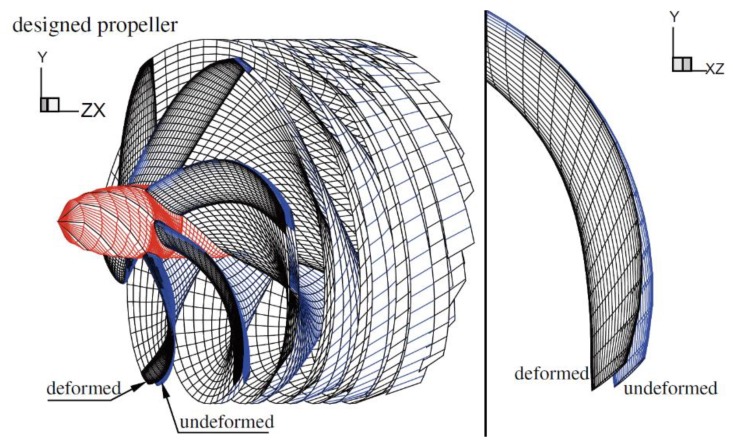
Undeformed and deformed geometry of a marine turbine with bend-twist coupled composite blades for improved efficiency [8].

Bend-twist coupled composites are most often used as the primary materials of marine [8,99,100] or wind turbine blades [101]. Mortley et al. [8] demonstrated that a marine turbine with bend-twist composite blades can work significantly more efficiently under most circumstances compared to a turbine with rigid blades (Figure 7). Coupled composites can not only make structures more efficient, but can push their applicability limits further, too. Shakya et al. [101], investigated the effect of bend-twist composites as the skin material of wind turbine blades. They found that they were able to increase the critical flutter speed of a turbine by 100%.

Using composites may not be the only way to achieve advanced mechanically actuated shape changes, but their outstanding specific mechanical properties and great tailorability of their mechanical behavior make these materials one of the most interesting and most promising candidates when it comes to developing more and more efficient structures, which is without a doubt the tendency in today’s industry.

Table 3 contains information about some mechanically actuated shape-changing concepts.

## 5. Applications

So far, we have discussed a variety of different literary approaches to achieve non-conventional deformations. In this section, we present some actual applications of morphing structures and materials to show their industrial value.

More advanced Ford Mustang models use an active damping (suspension) system instead of a traditional one. The non-conventional deformation occurs on the microscale, where magnetic particles align themselves in lines when exposed to a magnetic field. This increases the apparent viscosity of the fluid, leading to increased stiffness of the damper. The change in viscosity happens in a few hundredths of a second and is reversible just as quickly. This way the car can adjust the stiffness of its suspension according to different driving situations (e.g., cornering), which results in a smoother and more efficient ride [102]. 

NextGen Aeronautics developed an aircraft capable of significant in-plane wing morphing to adapt to different flight scenarios. Multiple linear actuators modify the shape of the flexible elastomeric wing skin to achieve the complex deformations, and wind tunnel and even flight tests proved the functionality and positive aerodynamic effect of the system [103]. 

Thermally actuated deployable structures, such as SMP based systems, are great alternatives to conventional, heavier solutions for space applications. NASA, for instance, developed a 35-meter diameter radar to monitor weather anomalies (e.g., hurricanes) from space, but deploying such a huge structure requires novel solutions, for instance combining shape memory and inflatable structures to deploy the space radar [104].

In 2018, the Fraunhofer Institute for Wind Energy Systems (IWES) started the testing of bend-twist coupled composite wind turbine blades. The 20-meter long smart rotor blades are designed to adapt to the changing environmental conditions (i.e., wind speed and direction). This example shows that the concept is already at the level of industrial application [105].

**Figure 8:**
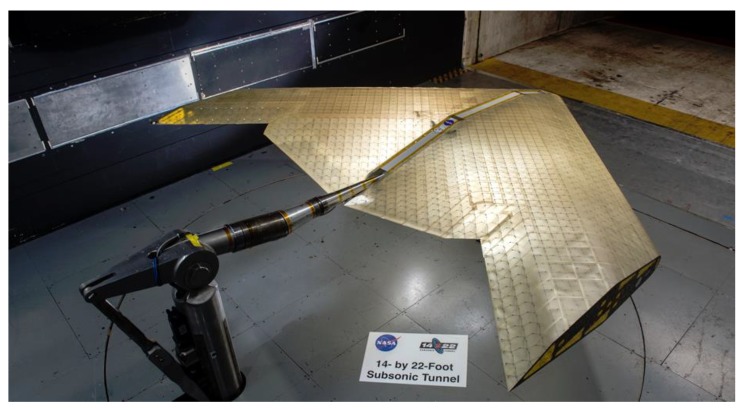
Passively morphing aircraft capable of shape adaptation to different flight conditions [106].

In 2019, MIT and NASA engineers developed a wing assembly capable of passive shape changes in response to changing flight conditions (i.e., mechanical loads). The researchers emphasized that the structure shows shape changes similar to what was already possible previously, but now morphing is passive. This means a smart structure that operates more efficiently under a variety of conditions than its conventional counterparts. The wing is an assembly of a large number of components of different stiffness forming a lightweight lattice framework covered by a polymeric skin. Due to the clever design of the structure, it behaves as a metamaterial on the structural level. It is both rubbery and stiff with extremely light weight and shape adaptation capabilities (Figure 8) [106].

Although numerous other examples could be presented here for the industrial application of morphing materials, the selected concepts sufficiently demonstrate that materials and structures with non-conventional shape changes offer advanced solutions even at the highest levels of engineering (e.g., aerospace). Also, it is important to identify the applicability of certain morphing concepts regarding the scale of the desired shape change. Usually, piezo-electric actuation is more suited for the micro applications, while motorized concepts can be used on the larger scales, for instance. Taking other considerations into account (e.g., static or dynamic actuation, actuation force, etc.) too, the optimal actuation concept is always dependent on the application scenario.

## 6. Summary and Outlook

**Figure 9:**
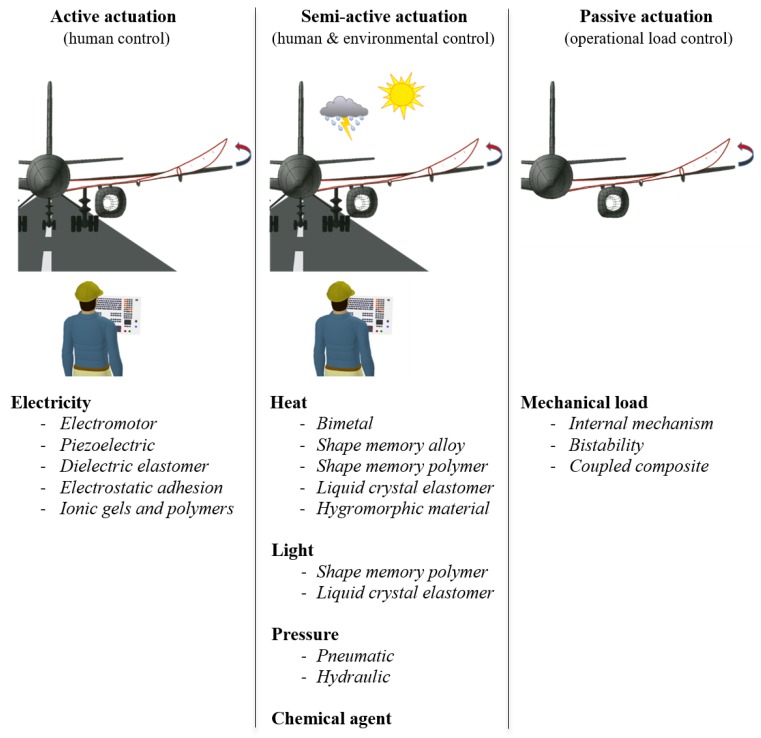
Schematic abstract of approaches to achieve non-conventional shape changes with.

Non-conventional shape changes can be actuated in various ways (by electricity, heat, pressure, etc.), and numerous different concepts exist for each kind of actuation (Figure 9). 

Electromotors provide good controllability when deforming structures, therefore they are widely used in the industry, however the weight they add to any system is a significant disadvantage. Weight reduction and efficiency improvement are some of the main driving forces of research aiming to develop materials that demonstrate shape changes other than conventional deformation (e.g., extension in response to tensile force or bending in response to bending moment). Electrosensitive materials (e.g., piezoelectric materials or electroactive polymers) can convert electricity into vibration, bending, twisting, etc. Generally, the main advantage of electro-actuated materials is their outstanding controllability almost independently of working conditions. Heat-actuated shape changes are most often achieved with shape memory polymers or alloys, but other concepts, such as liquid crystal polymers, are also researched. A common limitation of these systems is their often relatively low maximum actuation forces, but the large strains are capable of justifying their application in many cases. Pressure-actuated systems usually use inflatable pressure chambers for actuation, with which they can produce even complex deformations. However, the application of pressure is sometimes a challenge. Besides electricity, heat and pressure, light, chemicals and mechanical loading are commonly exploited to actuate non-conventional shape changes. However, mechanical actuation is by far the most researched area. The main advantage of mechanical actuation is its passive nature, as structural parts are inevitably under load during operation. This way, the system might not need any additional energy for actuation. On the other hand, this is one of its main limitations, since deformation cannot be controlled entirely independently of operating conditions. Composites have possibly the greatest potential as mechanically actuated morphing materials as they combine great tailorability of mechanical coupling behavior (e.g., bend-twist or extension-twist coupling) with outstanding specific mechanical properties. Coupled composites can make structural parts work more efficiently, especially where the optimal shapes under different loading conditions are known (e.g., wind turbine blades). Because of their industrial significance, it is expected that coupled composites will be the main focus of projects developing morphing materials in the near future. Although several publications describe the design process and performance of such composites, more emphasis has to be placed on how to overcome manufacturing challenges, which are mainly due to the (often) asymmetric layup of coupled composites. To exploit the morphing capability of composite materials, more research projects need to focus on the understanding of the mechanics of asymmetric layups and the contribution of asymmetry to hygro-thermal warping. This will hopefully serve as a good basis for designing stiff and strong shape changing materials and structures without the disadvantages of warping. The aerospace, wind energy and several other industries could greatly benefit from these results.

## Figures and Tables

**Table 1:** Electro-actuated morphing systems.

Working Principle	Deforming Material/Structure	Deformation Mode	Testing	Modelling	Measurement Results, Comments	Reference
Torque rods connected to servo motor	Flexible membrane wing	Twist	Flight tests: roll control	Linear time-domain model approximated from flight data	Good vehicle controllability: roll maneuvers carried out by morphing (primarily).	[9]
Torque rods connected to servo motor	Flexible membrane wing	Twist	Wind-tunnel and flight tests: roll control.	Numerical model (structural and aerodynamic) and genetic algorithm-based optimization for improved roll control. Good correlation with test results.	34% wing maneuverability and 6% wing efficiency improvement over baseline, but there is a trade-off between the two properties.	[10]
Servo motor moving loading rod up and down	Carbon fiber reinforced wing skin	Camber	Genetic algorithm-aided wind-tunnel tests: camber-lift relationship. 3 attachments to skin: two fixed attachments and a motorized attachment in between.	Hardware-in-the-loop optimization (see testing).	Optimized wing camber for maximum lift or efficiency by means of morphing actuated by a single servo motor.	[11]
Electromotor shears pivotally connected bars	Pivotally connected bars (in a rectangular layout) with fiber mesh and matrix material enclosed	In-plane shearing (not exclusively)	Temperature controller changes the elastic properties of the matrix (heating softens the matrix) and then the motor deforms the structure. Shape is stabilized by subsequent cooling.	-	Improved airfoil adaptability for better efficiency by significantly changing its shape and size.	[12]
Single electromotor drives complex motions of connected segments	Segments consisting of solid circular disks, Bowden cables and soft rubber skin	Peristaltic locomotion	3 segments controlled by a single motor. 3 tests: moving on flat floor, and through a tunnel with or without a payload	Analytical calculations to translate motor rotation to linear movement of Bowden6 cables	Comparable peristaltic locomotion results to a worm robot with multiple actuators.	[13]
Piezoelectric actuation integrated in sandwich wing structure	Sandwich structure of a wing trailing edge	Camber	Static deflection and load test; dynamic actuation test	3D finite element model with piezo behavior	25 Hz bandwidth and ±15° deflection	[15]
Piezo-composite actuator integrated in wing structure	Glass-epoxy skin	Camber/pitch rotation angle	High voltage current on 2 actuators for camber morphing and 1 actuator for rotation	Analytical rotation and bending angle, curvature and bending stiffness calculations. Greater predicted deformation-nonlinearity as a function of excitation voltage than test results, but close values.	450V: 1.4° camber 5.4° pitch rotation angle	[16]
Piezoelectric actuator integrated in wing structure	Carbon and glass fiber composite skin	Camber	-	Numerical aeroelastic (structural and aerodynamic) optimization	Up to 1100V: Optimized actuator-skin thickness ratio for maximum roll control and minimum weight. Piezo actuators provide not only energy for morphing but stiffness for the skin, too.	[17]
Piezoelectric actuator for vibration (and shape memory alloy for static deformation)	A section of a wing structure with integrated actuators	High-frequency vibration (piezo) and static camber (shape memory alloy)	Wind-tunnel testing: drag and lift measurements for more than 3000 configurations	2D finite element model for vibration frequency and static displacement	Wind tunnel tests: lift-to-drag ratio improved by 16% because camber and vibrating trailing edge improved drag and lift by a few percent.	[18]
Piezoelectric actuation (with thermopolymer and shape memory polymers)	Elastomer wing skin	Folding/sweeping	Full-scale subcomponent tests for each type of actuators	Finite element analysis (to evaluate drag)	Significantly modifiable wingspan and wing area during flight making the plane suitable for multiple purposes (e.g., cruising or high-speed dash)	[19]
Dielectric elastomer actuator (DEA)	Elastomeric polymer film	Compressed thickness–expanded area	Films coated with electrode material on both sides—electrostatic field compresses material	Analytical model—effective pressure derived from voltage	Silicone elastomers: 117% strain Acrylic elastomers: 215% strain Good energy efficiency (more than 80%) and swift response speeds (up to 2000 Hz)	[24]
Dielectric elastomer actuator (DEA)	Acrylic elastomer with carbon nanotube electrodes	Contraction	Weight on relaxed actuator. When the electric current is applied, the actuator contracts and lifts the weight.	Energy density–electric field function agrees with experimental results.	Measured energy density (19.8 J/kg in response to 3.5 kV) close to maximum human muscle potential.	[25]
Electrostatic adhesion	CFRP sandwich with PVC core	Bending (resistance)	High-voltage current on copper-polyimide electrodes placed between the split halves of the PVC core.	Analytical calculations and numerical modelling	4 kV voltage applied: 112% increase in bending stiffness for the sandwich test specimen	[27]
Electrostatic adhesion	CFRP sandwich with carbon-filled rubber core	Bending (resistance)	Quasistatic cantilever bending test where bending stiffness was modifiable by the electricity applied	Analytical model based on solid–solid friction interaction	18-fold change in bending stiffness	[28]
Electrostatic adhesion	Polyurethane core with interlocking (cosine wave form) copper-polyimide electrodes between the core halves	Change in shear force (and therefore bending stiffness)	Shear test	-	The reversible adhesion for non-planar electro-bonded laminates is similar to the simple, planar ones (about 7-10 kPa shear stress before slipping at 2 kV) with the added benefit of anisotropy due to the orientation of the electrode	[29]
Electric mechanical switches	Sandwich structure with truss core where the trusses are mechanical switches	Change in thickness, transverse shear and bending	Tensile tests (internal shearing) of an individual mechanical switch.	Analytical shear strength calculations. Calculated maximum transferable force is one order of magnitude higher than the measured force.	2000V: 1.3 N measured threshold force before the aluminum electrode starts to slip out (max. transferable force).	[30]
Electrostatic adhesion	Polyimide with nickel electrodes	Bending (resistance)	High-voltage current on Ni electrodes stacked with polyimide layers and then bending moment applied (3-point bending)	Analytical bending force calculations for stacking design. Difference from test results originates from the neglected interlaminar friction at zero voltage.	The applied voltage squeezed layers together leading to increased interlaminar friction and bending stiffness.	[31]
Ionic conducting polymer	Electrochemically deposited polypyrrole-coated nylon 6/6 micro-ribbons	Bending/twisting	Cyclic voltammetric measurements of free-standing micro-ribbons (-0.6V to +0.6V)	-	Significant twisting with a response time of ~5 s (0.6V) for the single-side-coated ribbons. (Temperature and pH activity tested as well)	[33]
Ionic gel	Bucky gel (tri-layer gelatinous mixture of electrolyte film, carbon nanotubes and ionic liquid)	Bending	Electrical current (2V) to actuator while measuring blocking force	Two-carrier model for back-relaxation of Bucky-gel	Maximal blocking force of 2 mN with back relaxation after a few seconds due to the relocation of anions and cations	[35]
Electro-responsive hydrogel	Hydrogel with aluminum hydroxide nanoparticles	Bidirectional bending	Dyed hydrogel strip placed between two Pt electrodes in Na_2_SO_4_ solution. Bending due to applied electricity.	-	Bidirectional bending, cyclic actuation (10 times) and high tensile strength of 2 MPa were demonstrated	[37]
Electro-origami/electro-ribbon	Steel strips insulated with PVC tape	Closing separate strips	Cyclic stimulation testing (100,000 cycles)	Analytical interfacial (attraction) force calculations	Electro-ribbon actuator lifts thousand-fold its weight and contracts by more than 99% with a specific energy similar to the specific energy of human muscle	[40]

**Table 2:** Heat-, light-, pressure- and chemically actuated non-conventional deformations.

Working Principle	Deforming Material/Structure	Deformation Mode	Testing	Modeling	Measurement Results, Comments	Reference
Bimetal (thermal)	INVAR (low CTE) and B72M (high CTE) alloy curved bimetal strip	Bending and snap-through	Power management circuit (energy harvester, DC-to-DC converter, storage). Measuring electric output power.	Circuit modelling and analytical calculations for output power	More than 13 µW output power for a 0.26g bimetal with 3 cm^2^ surface when placed on a 60°C hot source	[42]
Shape memory alloy (thermal)	NiTi coiled spring	Contraction (spring effect)	Weight placed on free-hanging spring (in martensite phase) and then heat applied for contraction (change to austenite phase). Displacement measured.	Effective displacement calculations	500 mm long coil with 400 µm diameter: more than 1000 J/kg energy density and 50% contraction	[6]
Shape memory polymer (thermal)	Multiblock copolymer (switching and hard segments)	Regaining original shape	Cyclic thermomechanical tests (straining, fixing, applying heat to recover)	-	Strain fixing capability up to 99.5% and strain recovery rate approaching 100% depending on the number of cycles (~80% in the first cycle)	[48]
Shape memory polymer (thermal)	Multiblock copolymer	Regaining original shape	Thermomechanical and sol–gel tests (spectrometry and NMR for chemical structure)	-	807% recoverable strain	[49]
Shape memory polymer composite (thermal)	Multi-walled carbon nanotube-filled polyurethane SMP	Regaining original shape	Voltage applied to samples and shape change monitored (FT-IR for reactive group investigation and DSC for transition temperature)	Calculation of the efficiency of energy conversion	Increased stress and modulus at 100% elongation but decreased failure strain compared to the unfilled SMP. ~10% efficiency of energy conversion	[53]
Liquid crystal elastomer – LCE (thermal)	Micron-sized LCE pillars	Contraction	Heating individual LCE pillars and investigating their contraction under a microscope	-	300% to 400% recoverable contraction	[56]
Hygromorphic material (thermal)	Patterned trilayer film (PpMS/PNIPAM/PpMS)	Origami (localized swelling)	Origami structure submerged into heat-transfer liquid and heated from 22 °C to 55 °C (and cooled back). Shape change investigated in thermal equilibriums	Folding angle calculations	Reversible complex-shaped self-folding origami	[58]
Shape memory polymer (light)	Polydopamine nanocoated SMP	Regaining original shape	Photo-responsiveness is measured with a dynamic mechanical analyzer (DMA) while keeping the strain fixed	Finite element modelling (mechanical) to simulate the nanocoat pattern-bending behavior relationship	Efficient photoactuation performance without worsening the original functionality or mechanical properties of the heat-actuated SMP	[61]
Liquid crystal elastomer (light)	LCE sheet with azobenzene	Bending	Dual circuit test setup: bending LCE sheet closes circuit and illuminates a LED lamp. One side bending to UV, opposite side bending to humidity.	-	Successful demonstration of a touchless electronic device actuated by either humidity or UV light resulting in opposite bending responses.	[79]
Pneumatic (pressure)	Wing structure with aluminum rib sections covered with latex sheet	camber	Wind tunnel-load cell balance test to measure lift and drag	-	Up to 10% change in camber	[80]
EFA-balloon (pressure)	Elastic polydimethylsiloxane (PDMS) cylinder with eccentric, pressurizable internal void	bending	Statically pressurized cylinders with different void eccentricities and radii	Analytical Euler–Bernoulli calculations (not accurate at large deflections) and finite element analysis for cross-sectional deformations and stresses	Example result: 0.14 mm^−1^ curvature at a pressure of 0.1 MPa (10 mm length, 0.5 mm diameter, 0.09 mm eccentricity and 0.35 mm inner void radius)	[72]
Hydraulic balloon (pressure)	Elastic polymer with flat bottom and folded microchambers on top (longitudinal section)	bending	Pressurizing the water in the membrane with a syringe and measuring bending deformation	-	Up to 180° bending deformation of micro-scale actuators	[68]
Pneumatic balloon (pressure)	“Microhand fingers” (4 mm long) consisting of silicone blocks connected by inflatable balloons	bending	Pressurizing balloons with compressed air	-	Full closure of microhand fingers at 240 kPa pressure	[7]
Pneumatic (pressure)	PDMS sheet with patterned pressurizable voids on two levels	torsion	Pressurizing two sets of voids (left and right sides of the actuator) on the opposite sides of the neutral axis. Opposing bending moments lead to twisting. Torsional angle is measured at different pressures.	Analytical torsion angle calculations	6.5° torsion for every mm along the length of the actuator (7 mm wide, 0.65 mm thick) at 178 kPa	[74]
Chemical agent	Poly(vinyl-acetate) (PVAc) with percolating cellulose nanofiber system	- (change in stiffness)	Pouring water (as the chemical agent for competitive hydrogen bonding) to the PVAc with cellulose fibers and measuring modulus	-	A stiffness change of 3 orders of magnitude (from 5 GPa to 5 MPa) due to dominant water-cellulose hydrogen bonding compared to cellulose-cellulose hydrogen bonding	[76]

**Table 3:** Mechanically actuated non-conventional deformations.

Working Principle	Deforming Material/Structure	Deformation Mode	Testing	Modeling	Measurement Results, Comments	Reference
Internal mechanism (translation)	Span-wise spars translating closer to or further from each other in wing box	(Resistance to) twisting	-	Quasi-static aeroelastic suite developed in MATLAB to model the adaptive torsion wing (plus FE model)		[82]
Internal mechanism (clutch)	Span-wise discontinuous internal walls fixed or released by a simple clutch system	(Resistance to) twisting	Measuring rotation as a function of applied force and wall displacements (fixed with clutches)	Analytical torsional stiffness calculations (Euler–Bernoulli, Saint–Venant, etc.)	Linear correlation between wall displacement and torsion, and significantly modifiable shear center for further torsion control	[85]
Bistability	Symmetric (prestressed) carbon fiber reinforced composite laminate	Snap-through between bistable states (bending and twisting)	Prestressing certain laminae prior to curing. Measuring snap-through loads after curing.	Analytical and finite element modelling of prestressed laminate bistability	Up to 72% increase in snap-through loads compared to asymmetric (and non-prestressed) bistable laminates for a 4-ply composite. The reduced hygrothermal variability of prestressed symmetric composites compared to asymmetric ones is another advantage.	[87]
Bistability	Rotor blade flap consisting of symmetric prestressed carbon fiber reinforced composite laminates	Snap-through between bistable states (bending deflection)	Bending moment applied to the laminates on a tensile machine while measuring force and deflection.	Analytical calculations for deflection and work (plus numerical simulations)	10° snap-through deflection of the bistable flap. The snap-through load and deflection are tunable by the number, layup, material properties and placement of the laminates and by the applied prestressing.	[90]
Coupled composite	Carbon-epoxy composite box beam	Twisting to extension	Beam tests for validating the finite element analyses	Dynamic aeroelastic finite element analysis of the “self-twisting” rotor blade	20% maximal improvement of lift-to-drag ratio compared to uncoupled blades	[97]
Coupled composite	Carbon-epoxy composite marine propeller	Twisting to bending	Cavitation tank tests of a propeller with a 200 mm diameter rotated at 7-20 rps (3.55 m/s axial fluid speed). Deformations observed, thrust and torque measured.	Genetic algorithm-based layup sequence optimization and numerical simulation of the bend-twist behavior	Propeller with optimized layup passively changes its pitch angle to changing ship speeds, leading to improved torque curve and efficiency. Numerical results were difficult to compare to experimental results because of small deflections and vibrations.	[99]
Coupled composite	Carbon-epoxy composite marine propeller	Twisting to bending	-	Numerical simulations for pitch angle, working efficiency, thrust and power comparing the bend-twist propeller with a rigid one	Bend-twist coupled propeller works significantly more efficiently compared to its rigid counterpart in most cases.	[8]
Coupled composite	Fiber reinforced composite wind-turbine blade with asymmetric skin layup	Twisting to bending	-	Aeroelastic (structural and aerodynamic) modelling of the mechanical behavior of the composite blade	Critical flutter speed of the blade increased by 100%. This was achieved by using bend-twist coupled composites in the skin instead of regular (symmetric) layup.	[101]
